# Observations on the Efficacy of the Salts of Silver in the Treatment of Secondary Syphilis

**Published:** 1838-10-24

**Authors:** William P. Johnston, James Trudeau

**Affiliations:** Georgia; Louisiana


### FOREIGN CORRESPONDENCE.

*Observations on the Efficacy of the Salts of Silver in the Treatment of Secondary Syphilis, by* William P. Johnston, M. D., *of Georgia, and* James Trudeau, M. D., *of Louisiana.*

In 1813, Mr. Serre, of Montpelier, published a short essay, in which he recommended the salts of silver in the treatment of venereal affections. Many of the French physicians, among whom we may mention Mr. Cullerier, Surgeon to the Venereal Hospital of Paris, have tried these preparations in a number of cases, and though they do not indiscriminately recommend this treatment, are still disposed to acknowledge its partial efficacy. During the past year, Mr. Biett has himself been experimenting with the silver, in several cases of secondary syphilis. Previous to giving the cases, which we have collected in his wards, relative to this mode of treatment, we will briefly review the essay of Mr. S., which may not yet have reached our country. The essay contains twenty-five cases. Of these, there were seventeen of the primary, and eight of the secondary form of syphilis. Of the former, including chancres, phymosis, buboes, and gonorrhœa, the exact duration of the disease is given in eight cases only, the average of which was more than two months. In the remaining nine of the primitive form, Mr. S. has either neglected to inform us of the date of the entrance of his patients, or the date of their discharge, so that it is impossible to say how long the patients were under treatment. In one of the above cases the duration was five months; the patient was first treated by the chloride of platina, and afterwards by the chloride of silver; but we are not informed how long either the one or the other was continued. We cannot, therefore, in this case, appreciate the effect of the silver. The twenty-third observation presented chancres on the penis and scrotum; this, Mr. S. considered as a case of secondary syphilis, because the patient declared he had not exposed himself for eight months previous. But, it seems to us, the description given by Mr. S. differs in nothing from an ordinary case of primary chancres, and hence we have included it in this class. This patient took seventeen grains of the chloride of silver, and eleven grains of the bichloride of mercury, before a cure could be effected. The number of cases of primary syphilis treated by the salts of silver contained in the essay of Mr. S., are, we think, too few in number, and the history too imperfect, to enable us to arrive at any correct conclusion, as to the real efficacy of silver in the treatment of this form of the disease. But we cannot but think that a remedy that requires an average of two months to cure an ordinary chancre or a bubo, is not deserving much consideration.

Of the eight cases of secondary syphilis, the first (case No. 8) exhibited condylomatous tumours around the anus, with ulceration of the fauces. The patient was discharged cured; duration of treatment not given. In the second, (case No. 15,) the patient's body was covered with lenticular pustules. He was discharged, still exhibiting traces of the disease; duration of treatment not given. In the third, (case No. 16,) there were chancres, exostosis, pains in the bones. The patient was discharged after a certain time, the exostosis remaining the same as at his entrance; duration of treatment again not given. The fourth, (17th case,) exhibiting vegetations of a secondary form around the anus, was cured in thirty days. The fifth, (case No. 19,) had copper coloured spots covering the body. In this case, the silver was at first administered, and failed. The cure was effected by the bichloride of mercury. The sixth, (case 21,) secondary ulceration of the glans penis, cured; duration not given. The seventh, (case 24,) was affected with spots, of various sizes, covering the whole of the body. The oxide of silver was given, until the patient had taken as high as sixteen grains. The bichloride of mercury and sudorific drinks were then administered. The patient improved under this treatment, but was discharged before a cure was completed. The eighth, (25th case,) had pustules upon the scrotum and penis, with ulcerations of the foot. The patient was discharged, cured, after two months of treatment. In four months the disease re-appeared, and he was obliged to enter another hospital. Of these eight cases, the bichloride of mercury was resorted to, after the silver had failed, in two cases. In a third, the silver had no effect upon the exostosis. The fourth was discharged before a complete cure was effected. In a fifth, the disease reappeared. The sixth, consisting merely of an ulceration of the glans penis, was cured, but duration not given. Why Mr. S. considered this as secondary syphilis, we cannot say; he ought, perhaps, to have classed it among those of the primitive form. The remaining two, which, by a remarkable coincidence, prove to have been the mildest of the cases detailed, were cured; one in thirty days,—in the other the duration is not given.

After this success, Mr. Serre came before the public, recommending a substance as yet untried, at least in modern times, in the treatment of venereal affections, appealing to his cases as an evidence of its happy influence, both in the primary and secondary forms. Had the subjects of his observations been men of depraved constitutions and given to excesses, or accustomed to a nutrition at once scanty and unwholesome, we might perhaps have thought, with him, that two months was but a short time to cure a chancre or a bubo. But so far from this being the case, we are told that his patients belonged to the army; and as he does not declare to the contrary, we think we are entitled to believe that they, like the rest of the French army, received a good and wholesome nourishment, and were men of good constitutions.

Before dismissing the essay of Mr. S., we will briefly describe the mode and forms in which the silver was administered. Frictions on the tongue were the most usual method, and that to which he gives the preference. Pills were given in a few cases only, and an ointment was sometimes applied locally. For frictions, he followed the rules prescribed by Dr. Chrestian, in his jatralyptic method; that is, mixed with a large quantity of inert powder. The dose depended upon the preparation employed. The chloride of silver, and the chloride of silver and ammonia, which he regards as the most effectual preparations, were given,—the first, in doses of one-twelfth of a grain; the second, one-fourteenth of a grain. The cyanide and iodide of silver were prescribed in doses of one-eighth of a grain. Oxide of silver, and silver in substance, from one-quarter to one-third of a grain. The whole quantity administered in a single case varied from five to seventeen grains, according to the preparation given and the obstinacy of the disease. Mr. S. recommends the salts of silver, in higher doses than those which he employed. It is to the smallness of his doses that he attributes his failure in two cases. As to the particular action of silver on the economy, Mr. S.'s experiments have thrown no light. There does not appear to have been any alteration in the secretions or the circulation. In fact, there is no symptom noticed as following the administration of this remedy, except a slight diarrhœa, or colicky pains, which occurred in a few cases only, and which he attributes to an epidemic influence then prevailing. It is scarcely necessary to mention, in conclusion, the various advantages which Mr. S. considers his remedy to possess over mercury, such as its not producing salivation, or any of those effects upon the skin and bones which are so generally attributed to the latter.

To prove the inefficacy of silver, at least in the secondary forms, we will annex the condensed histories of a few of the cases, which we have referred to in the commencement of this paper.

*Hospital St. Louis*, (*Service of Mr. Biett.*)

PAPULOUS SYPHILIDE, (*Syphilide Papuleuse.*)

The subject of this observation, aged twenty-six years, was formerly a soldier. An illicit connexion, four years since, was followed by chancres on the glans penis. The patient put himself under the care of Mr. Lallemand, of Montpelier, by whom he was treated with the pills of Dupuytren,* (composed of guiacum, opium, and bichloride of mercury,) to which Mr. L. is particularly partial. The patient took two hundred and fifty pills, after which the chancres disappeared. Three years after, being at the time married, the ulcerations reappeared on the glans. The patient declares that he had not exposed himself to any cause which could have reproduced the disease. He entered the Venereal Hospital of Paris, and submitted to an antiphlogistic treatment. A cure followed. The patient entered St. Louis in the month of November, 1837. Shortly before his entrance, he was attacked by a violent periodical cephalagia, commencing towards sunset, gradually increasing for several hours, then declining and ceasing entirely towards day-light. A syphilitic eruption soon succeeded, and put a speedy end to this painful symptom. At his entrance, the eruption covered the whole of his body. He was immediately put upon the acetate of silver, in doses of one-quarter of a grain morning and evening. This treatment was continued for nearly three months, when, at the end of January, Mr. B. finding that with the exception of a considerable irritation of the alimentary canal, the patient's condition was the same as at his entrance, he discontinued the treatment, and immediately prescribed the proto-iodide of mercury. An improvement followed in a few days, when the patient becoming attacked with an angina which was at that time epidemic in the wards, a temporary cessation of the treatment became necessary. The improvement, however, which had been produced, continued. At the end of February, the patient, being cured of his angina, the proto-iodide was resumed, and on the 14th of April he left the hospital, perfectly cured.

*Remarks.*—This case presents one very important point, deserving notice. We refer to the nocturnal cephalagia, with which the patient was harrassed for some time previous to the developement of the eruption. The relief that was experienced in this case immediately after the appearance of the papules, leaves no doubt that the cause of the former was a syphilitic tint. That syphilis may imitate many of the neuralgic and other affections, is, in the opinion of many, as well established as that it imitates in a greater or less degree most of the diseases of the skin. But the difficulty of diagnosis is much greater in one case than the other. In the latter, aside from the history of the patient, we have a certain colour of the eruption, compared to that of copper, which at once points out the nature of the disease. In the former, we have no one characteristic symptom. It is true that violent neuralgic pains of a syphilitic origin are usually more or less periodical, and occur for most part at night. We would refer to the excellent treatise of Messrs. Trousseau and Pidons on Therapeutics, article Mercury, for the histories of some interesting cases on this point; one, a case of epilepsy, cured by mercury, after all other remedies had failed,—another, that of a banker of Paris, in whom the disease simulated gastralgia, with pains and vomitings, which appeared every evening. These symptoms, which had continued for ten years, entirely disappeared as soon as the patient became salivated. Other cases are noted of periodical

* Mr. B. says:—"These pills (Dupuyten's) ought never to be employed. The extract of guiacum which they contain renders them so hard that they not unfrequently pass entirely through the alimentary canal without being dissolved. Besides, the opium diminishes in a great measure the alterative power of the bichloride."

neuralgia likewise cured by mercury. All these patients had had one or more attacks of syphilis some time previous. We think that in cases of neurosis, especially where the paroxysms occur at night, and where other remedies have failed, the physician should resort to mercury, though there be no local evidence of syphilis, provided the patient has had this disease previously.

PAPULOUS SYPHILIDE, (*Syphilide Papuleuse.*)

The individual, of whom we shall now speak, is thirty-six years of age, of small size, and very muscular. In February, 1837, he was attacked with gonorrhœa and chancres around the corona glandis. He placed himself under the care of a quack, whose remedies produced no effect upon the disease. In March of the same year, he entered the Veneral Hospital, where he was treated by sarsaparilla and emollient drinks. He left this hospital on the 15th of April, and a few days after he was attacked with symptoms which Mr. B. designated as venereal, such as wakefulness, severe cephalagia, deep seated pains in the bones. These were mistaken for the premonitory symptoms of an acute disease. The patient was, in consequence, bled, purged, and placed upon low diet. On the 26th of March, 1838, he entered the St. Louis. The day after his entrance, a papulous eruption made its appearance over the whole surface of the body; upon which the general symptoms, mentioned above, which had continued until then, disappeared entirely. The patient was put upon the chloride of silver, in doses of half a grain, morning and evening. On the 22d of April, not the slightest change could be noticed in the eruption. The silver was now discontinued, and the proto-iodide of mercury, one grain morning and evening, was ordered. A change in the symptoms was soon produced, and the disease advanced steadily towards a cure. On the 30th of June, the patient left the hospital.

*Remarks.*—In this case, as in the former, we observe the eruption followed by an immediate cessation of the cephalagia. The chloride of silver was given in doses much larger than by M. Serre, and continued for twenty-six days, without, in any degree, affecting the disease. Nor could we observe that it reduced any action on any of the organs. The alimentary canal was not at all disturbed.

SYPHILIDE TUBERCULO-ULCÉRANTE.—(*Biett.*)

In consequence of a gonorrhœa contracted in 1831, an eruption appeared over the body of the patient, who is the subject of this observation. He was treated by his physician with the pills of Dupuytren. A cure followed this administration, but the disease reappeared in 1836. The patient then entered the Hôtel Dieu of Rouen, where he was treated by mercurial fumigations, which again effected a temporary cure. The disease again returned in a more aggravated form, without the patient having exposed himself to a fresh source of contagion. The disease continued increasing in severity until the 6th of April, 1836, when he entered the St. Louis. At this time, deep copper coloured ulcerations, with tubercles, covered his face, body, and legs. There was ulceration of the fauces, and the velum palati was almost destroyed. The disease, in a word, offered all the characters of the variety described by Mr. B., as "*Syphilide Tuberculo-ulcérante.*" There was, in addition to the above symptoms, extreme emaciation and very anxious expression of countenance; and, to such a state of despair was he driven by his disfigured and frightful appearance, that, as he afterwards declared, he had intended committing suicide, if his condition had not been speedily ameliorated. The cyanide of silver was commenced in doses of one-twelfth of a grain, gradually increased. Irritability of the alimentary canal, increased instead of the ulcerations, both in depth and surface, followed the administration of silver. Alarmed at the aggravated state of the patient, the silver was discontinued by Mr. B., and the watery extract of opium, in doses of half a grain, night and morning, ordered in its stead. This treatment was continued for a month, at the end of which time, the irritability of the intestines having ceased, the opium was replaced by the proto-iodide of mercury, one grain night and morning. Six days after the administration of this remedy, a favourable change was noticed in the appearance of the eruption, which continued advancing, without intermission, until the 16th of June, when the patient was discharged, perfectly cured.

*Remarks.*—In this case the silver was continued but a short time, in consequence of the diarrhœa and colic, which supervened. We have noticed these symptoms so frequently in the cases which have come under our observation, and Mr. B. cites in his Clinical Lectures so many others, that we cannot but regard them as being produced, in many instances, at least, by the direct action of the silver. The effect of the opium, in this case, was such as Mr. B. usually anticipates from its administration, namely, calming the irritation of the alimentary canal, and thus preparing the system for the proto-iodide of mercury.

TUBERCULOUS ULCERATED SYPHILIDE,

(*Syphilide Tuberculo-ulcérante.*)

This patient, aged twenty-nine years, contracted, in 1830, chancres, followed by buboes. He consulted a quack, by whom he was treated and cured. But the patient could not say what was the treatment employed. In 1831, an eruption appeared over his body; he continued, however, his occupation of "chiffonnier," until January, 1838, when he entered St. Louis. Upon his entrance, the septum narium had ulcerated away, and the ossa palati were in a state of necrosis. The face, shoulders, and a part of the anterior portion of the chest, were covered by tubercles of various sizes, ulcerated at their apex. The treatment was commenced with the phosphate of silver, in doses of one-twelfth of a grain, the doses being gradually augmented. No effect following, the phosphate was discontinued, and the chloride given in its place in the same doses. This treatment was continued until the month of March, at which time the whole amount taken of this preparation was thirty-eight grains; no change was produced in the disease. The patient was now put upon opium for a month. On the 1st of April the proto-iodide of mercury was ordered in the usual dose. A week after the administration of the first dose, Mr. B. pointed out a modification in the appearance of the ulcerations. These gradually assumed a healthy appearance, and became covered with thick crusts. In the month of June, the crusts commenced falling, leaving irregular and slightly rosy cicatrices, which finally assumed their usual whitish appearance. From being extremely emaciated, the patient acquired an ordinary degree of *embonpoint*. The cure was completed by the vapour baths. The whole amount of proto-iodide taken was sixty grains. On the the 7th of August the patient left the wards.

TUBERCULOUS SYPHILIDE, (*Syphilide tuberculouse.*)

This case was that of a man of temperate habits, aged about fifty-five years. In his youth he contracted a chancre, for which he used nothing but the local application of tobacco. In due time the chancres cicatrized; after which the patient continued free from any symptom of syphilis for the space of thirty-eight years; at the end of which period, an eruption appeared. The patient was at a loss to account for this, even when informed of its being evidently syphilitic, for he says that shortly after the chancres alluded to above had cicatrized, he married, and that he has had several children, all of whom have enjoyed good health, from their birth; and that he himself has led a correct and sober life, not having exposed himself anew since his marriage. At the entrance of the patient, small tubercles of a livid red hue about the size of peas, covered the whole surface of the body; but were more thickly grouped upon the face and shoulders. The cyanide of silver was immediately commenced, and continued for about a month, at the end of which period no change could be observed in the tubercles. Mr. B. was desirous of pushing this treatment still further, but an iritis supervening, it was discontinued for remedies in which he had more experience. Calomel and belladona were ordered, and, in a short time, the iritis disappeared; but a second iritis almost immediately after attacked the other eye. The same remedies were prescribed, and the same effect followed. Then a double iritis was noticed; still the same treatment was followed by the same effect. In less than three weeks from the administration of the calomel and belladona, the tubercles began sensibly to decline. In July there remained upon the body of the patient only some spots of a copperish colour, and some small cicatrices. The patient was then regarded as almost cured, when an iritis again attacked the right eye. The treatment with calomel and belladona was again resumed. August the 24th, the patient was entirely well of the affection of the eye. Scarcely any marks remained of the eruption which had existed, except the minute cicatrices noticed above.

*Remarks.*—This case presents many points to be noticed. In the first place, we remark that silver produced no change in the tubercles, but that calomel and belladona, given to combat the affection of the eyes, were soon followed by decided improvement. It is rare to observe such a succession of irites, as in the case of our patient. Another point of interest is the length of the period which elapsed between the primary and secondary forms; thirty-eight years of incubation will, to many, seem incredible. Whether the system may remain under the influence of a chancre for such a length of time, without betraying itself by one or more known symptoms, is a point difficult to decide, and one upon which we will not give an opinion. Mr. B.'s practice in cases exhibiting a secondary eruption, is to examine the penis carefully, and, if he finds upon it the cicatrix of a chancre, attended with a slight loss of substance, he attributes to it the secondary symptoms, without regarding the time which may have elapsed between the one and the other.

In the above cases, which we have been obliged to condense very much, it will be seen, that the salts of silver have invariably failed in producing the slightest modification in the disease. Mr. B., in his Clinical Lectures, says that he has employed the different preparations of silver recommended by Mr. Serre, in a vast number of cases, and that he has continued the treatment until the amount taken was thirty grains, without having ever seen it produce "beneficial effects, and in some cases to the manifest injury of the patients."

In conclusion, we will beg to say a few words of the remedies which, in the hands of Mr. B., have been so successful in the treatment of this form of disease. The proto-iodide of mercury, introduced by him in the treatment of syphilides, is that to which he gives the preference. It is given in the form of a pill in doses of one grain, with an equal quantity of lactucarium, night and morning. This remedy, in his hands, rarely produces salivation. He employs, in conjunction, the vapour baths, which he regards as a powerful agent in completing the cure and restoring the healthy functions of the skin. Opium plays an important part in the formulary of Mr. B. He employs it in cases where irritation of the stomach and bowels has been produced by the metallic preparation, or where the system generally is in a state of nervous irritability. His usual practice is to give one grain of the watery extract, morning and evening, during a month, and then he resumes the treatment with the proto-iodide. Mr. B. says he has often seen opium produce a decided amelioration in the disease, when all other remedies have failed. He has also remarked cases where a modification produced by the proto-iodide has continued under its use until a complete cure was effected. The liquor of Van Sweiten and sudorific drinks, are sometimes employed. Simple cerate is the only local application for the ulcers, and even this is but rarely used. In support of the treatment of Mr. B., which, from having long and carefully observed it, we can recommend, with the greatest confidence, we will quote the authority of Mr. Ricord. He says, "the preparation to which I now give the preference, not only in the treatment of the secondary, but also in that of the primitive symptoms, is the proto-iodide of mercury." Opium, he says, alone, or more frequently combined with other remedies, is one of the most useful agents in the treatment of this disease, and ought never to be neglected.

PARIS, September 18, 1838.

